# Supplementary material for: Constructing and Evaluating a Mitophagy-Related Gene Prognostic Model: Implications for Immune Landscape and Tumor Biology in Lung Adenocarcinoma
Source: Biomolecules. 2024 Feb 16;14(2):228. doi: 10.3390/biom14020228 (PMC10886790; doi:10.3390/biom14020228)
Supplement: Supplementary file 1 [file biomolecules-14-00228-s001.zip › biomolecules-2834382-supplementary.pdf]

**Table S1.** The mitophagy related genes extracted from GeneCards database classified by the relevance score

| Relevance score | Count | Gene symbol |
|---|---|---|
| > 5 | 15 | PRKN PINK1 MAP1LC3B VDAC1 FUNDC1 MFN2 SQSTM1 MAP1LC3A PHB2 ATG13 AMBRA1 ULK1 UBC TOMM20 USP30 |
| 4-5 | 10 | CLEC16A DNM1L RIMOC1 UBA52 MFN1 OPTN HUWE1 PGAM5 SLC25A4 RNF41 |
| 3-4 | 29 | ATG7 UBB SLC25A5 VPS13D TOMM40 VPS13C ATG4D TAFAZZIN ATG4B USP15 GABARAPL2 TOMM7 BNIP3L BECN1 ABCE1 CERS1 ATG5 TOMM70 BNIP3 TIGAR RHOT1 RPS27A LRBA ARFIP2 TOMM22 PHB1 FBXO7 RAB7A SPATA33 |
| 2-3 | 37 | MAP1LC3B2 MUL1 GABARAP CHUK ATG9A TP53 CISD1 FKBP8 CALCOCO2 CNOT4 CSNK2A1 TBK1 VDAC2 CSNK2A2 MTX1 ATG4A BCL2L13 SNCA MON1A PELO HDAC6 LRRK2 PRKAA1 MTARC2 PTRH2 BCL2L1 FIS1 OPA1 ATG14 TDRKH GBA1 ATG12 UBE2L3 MFF MAP1LC3C PI4KB SIRT1 |
| 1-2 | 181 | WIPI1 VCPNBR1 SPATA18 HTRA2 SESN2 ATG4C SAMM50 IMMT MAPK14 FANCC TBC1D5 STX10 STK4 NLRP3 ATAD3A MARCHF5 PLSCR1 BCL2 SRC CSNK2B TOMM5 MTERF3 TOMM6 MIEF1 BAG5 PEX13 MTX2 HSPA8 OGT RETREG1 MAVS PTEN CHCHD3 GABARAPL1 PARL TSC2 VDAC3 HK2 MAPK1 TSPO MAP2K1 FOXO3 MDH1 TBC1D15 OCIAD1 USP8 PI4K2A STOM KRT15 BLOC1S1 HIF1A ATM LRPPRC ATP1B1 APP BCAS3 PHAF1 ZFYVE16 BET1 GDAP1 WDR26 MCL1 LINC-PINT NR4A1 ALB HSPD1 HTT ATG16L1 TFEB NIPSNAP1 BAG6 CDC37 SIRT3 MST1 TRAP1 ATG2B TGFB1 NFE2L2 UQCRC2 NIPSNAP2 TFE3 VAPA DSP CAV1 TOM1 NME4 USP36 UBXN1 RMC1 RNF31 TCHP MTOR GPD2 SHC1 USP33 STX17 RNF121 CANX EPHA2 SLC12A6 ITCH SLC12A4 STEAP3 FADS2 UBE2G2 VPS35 ANO6 EMC1 LPCAT3 ARL6IP5 MON2 AMFR AKAP1 REEP5 SNX3 GDI2 CHMP2A ESYT1 ATG2A SPG21 RMDN3 SEC22B ARMCX3 TBC1D17 CCZ1B FLOT2 SH3GLB1 SREBF2 MIR155 PPARGC1A ALDH2 HSPA1L HMGB1 MAP2K3 LMAN1 FBXW7 ARIH2 |

| | |
|---|---|
| | ATP5IF1 MAP2K2 TIMM23 UBXN6 FTMT HSPA9 WIPI2 ZFYVE1 TIMM44 HRAS TFRC NDUFA10 NRF1 KRAS PRKAA2 AKT1 RCAN1 RB1CC1 USP35 TUFM PRPF8 BAX MAPK15 VIM MIR106B MIR93 MIR25 HK1 IGF1 NDUFV1 OSBPL5 RDH13 TAX1BP1 CKAP4 MRPS2 PRKCD BIRC2 HSPA1A TXN HNRNPM STOML2 ATAD3B LOC105378097 |
| **Total** | **272** |

**Table S2.** Analysis of the difference of mitophagy risk score in different clinic-pathological features

| Pathological | N | Risk score | PValue |
|---|---|---|---|
| Gender | | | |
| female | 270 | 7.904±1.107 | |
| male | 234 | 8.058±1.04 | 0.152 |
| Age | | | |
| > 60 | 336 | 7.919±1.096 | |
| ≤ 60 | 158 | 8.107±1.026 | 0.069 |
| Distant metastasis | | | |
| M0 | 335 | 8.025±1.126 | |
| M1 | 25 | 8.441±1.341 | 0.082 |
| Lymph node metastasis | | | |
| N0 | 324 | 7.841±1.04 | |
| N1 | 95 | 8.166±1.036 | 0.052 |
| N2 | 71 | 8.399±1.125 | **0.001** |
| N3 | 2 | 8.035±0.798 | 0.995 |
| Invasion depth | | | |
| T1 | 169 | 7.72±0.919 | |
| T2 | 269 | 8.04±1.053 | **0.011** |
| T3 | 45 | 8.287±1.392 | **0.007** |
| T4 | 18 | 8.489±1.428 | **0.019** |
| Stage | | | |
| Stage I | 270 | 7.765±0.991 | |
| Stage II | 120 | 8.075±1.07 | 0.056 |
| Stage III | 80 | 8.454±1.107 | **<0.001** |
| Stage IV | 26 | 8.432±1.315 | **0.014** |
| Smoking history | | | |
| Current | 119 | 8.108±0.99 | |
| Never | 72 | 7.711±1.105 | **0.034** |
| Reformed (≤15) | 128 | 7.705±1.091 | 0.985 |
| Reformed (>15) | 167 | 8.164±1.085 | **0.015** |

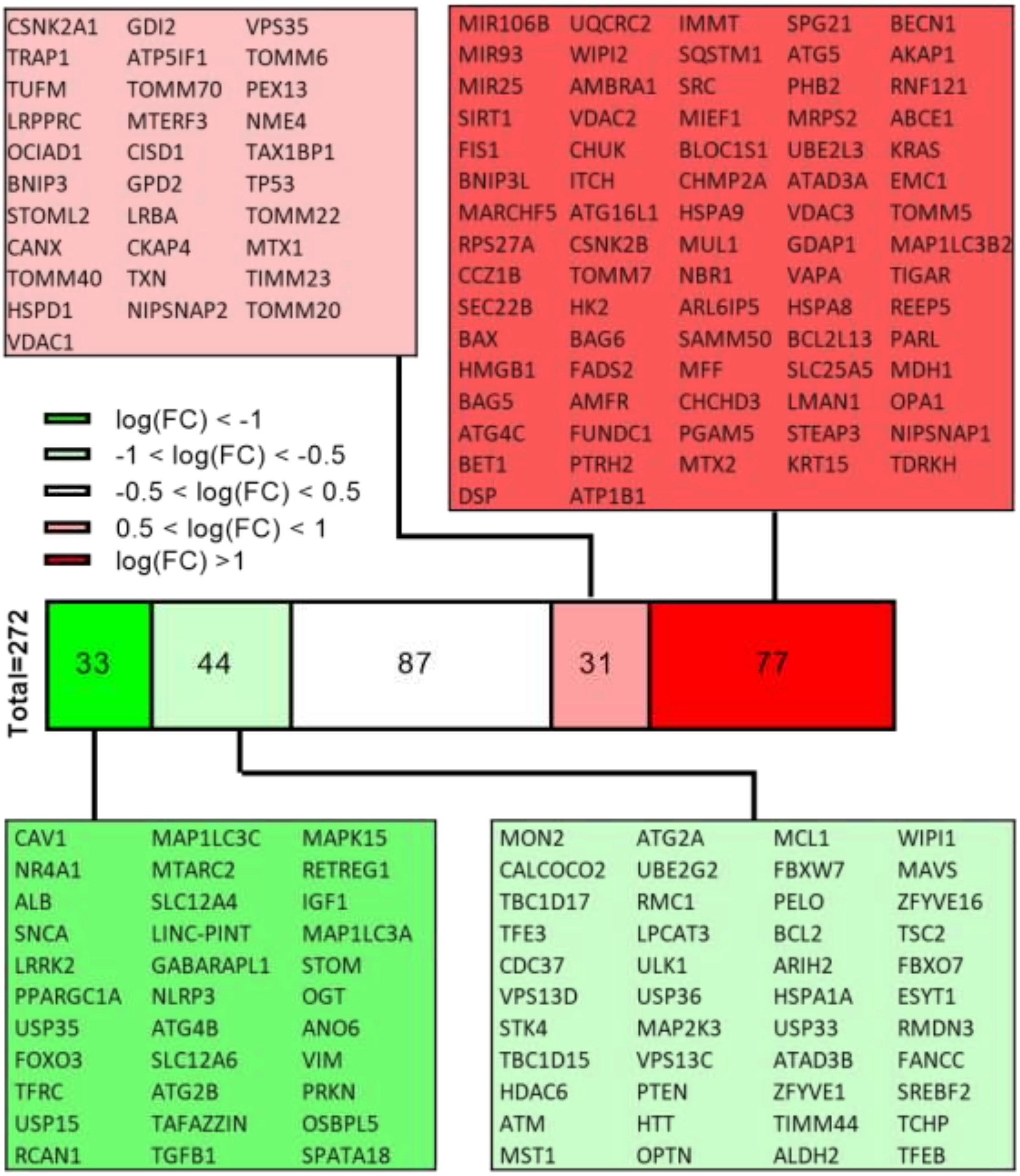


**Figure S1**. Different expression analysis of 272 MRGs in TCGA LUAD dataset.

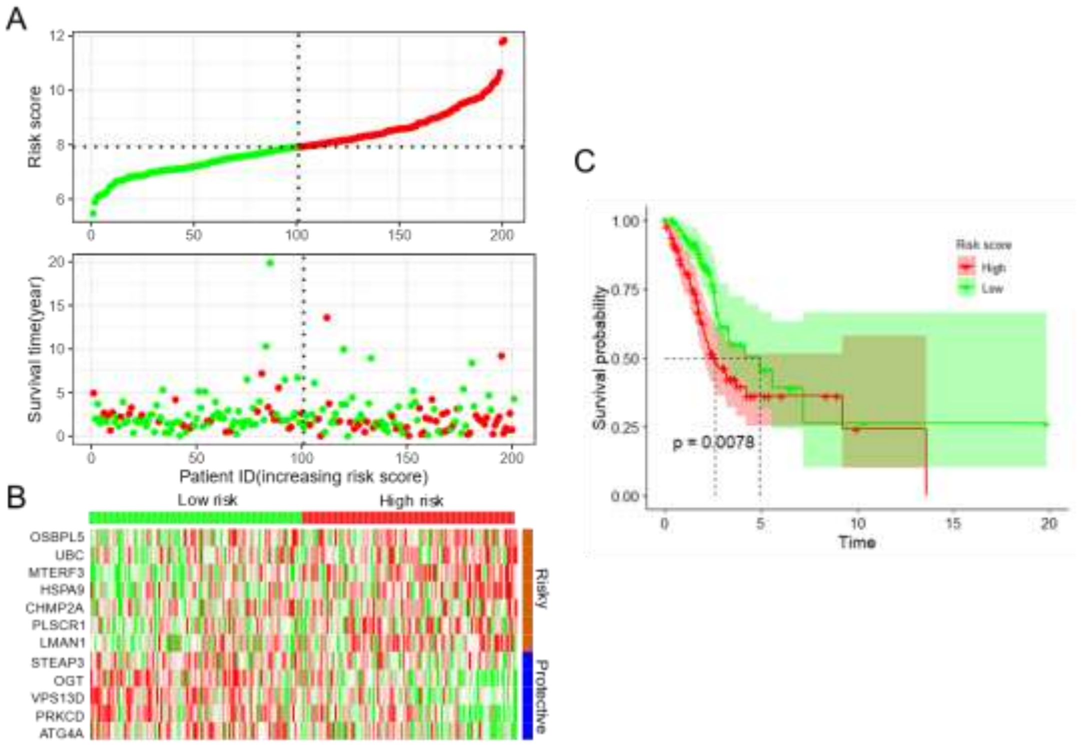


**Figure S2**. Validation of the prognostic model with 12 MRGs in internal testing set. (A) Risk score distribution and survival status of training group. (B) Heatmap shows the expression of 12 MRGs in training group. (C) Kaplan–Meier curve of overall survival in training group. MRGs: mitophagy-related genes.

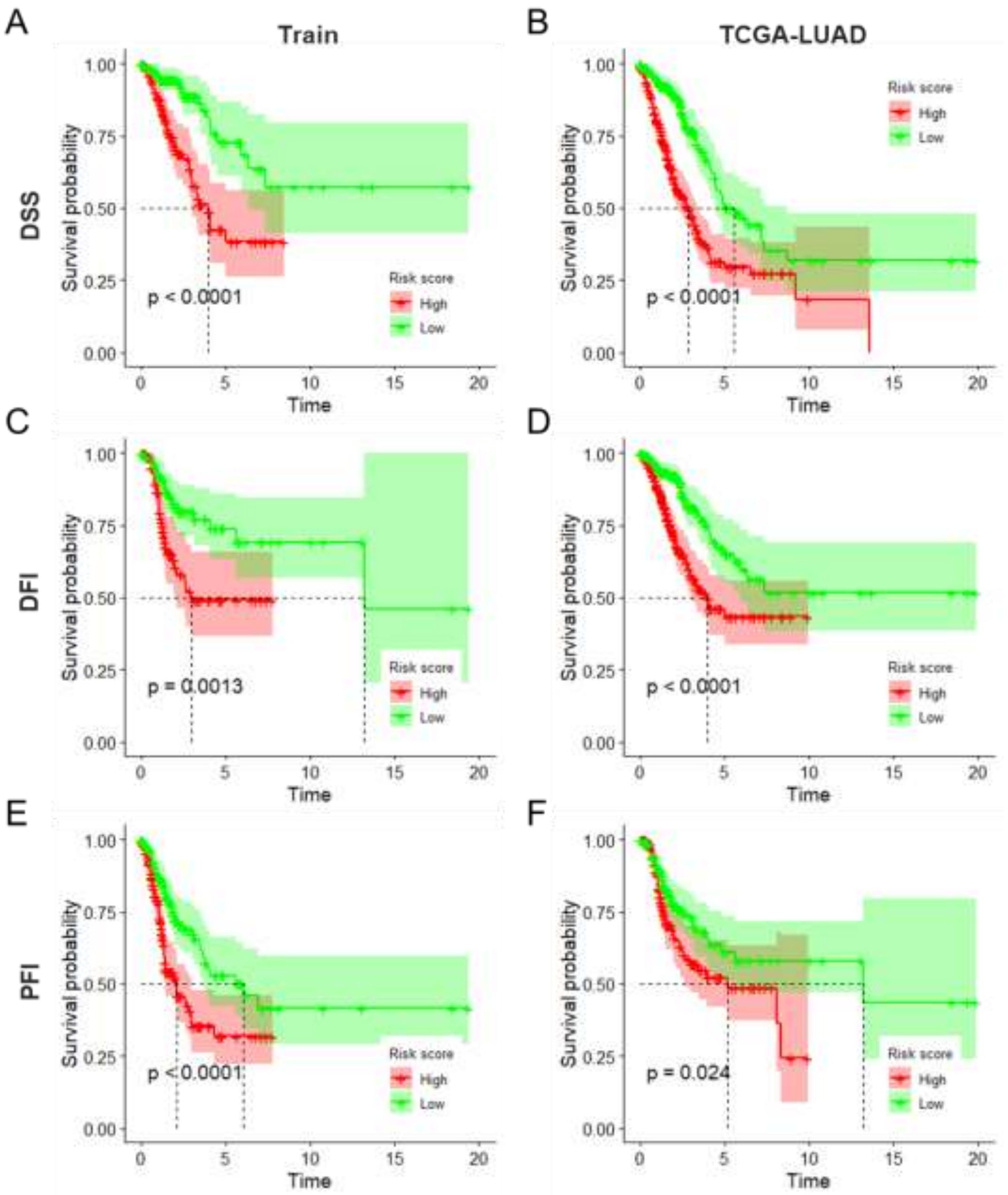


**Figure S3.** Kaplan–Meier curve of mitophagy risk score for different survival indicators. Kaplan–Meier curve of mitophagy risk score for DSS in training set (A) and entire TCGA-LUAD (B) dataset. Kaplan–Meier curve of mitophagy risk score for DFI in training set (C) and entire TCGA-LUAD (D) dataset. Kaplan–Meier curve of mitophagy risk score for PFI in training set (E) and entire TCGA-LUAD (F) dataset. DSS: disease specific survival; DFI: disease free interval; PFI: progression free interval.

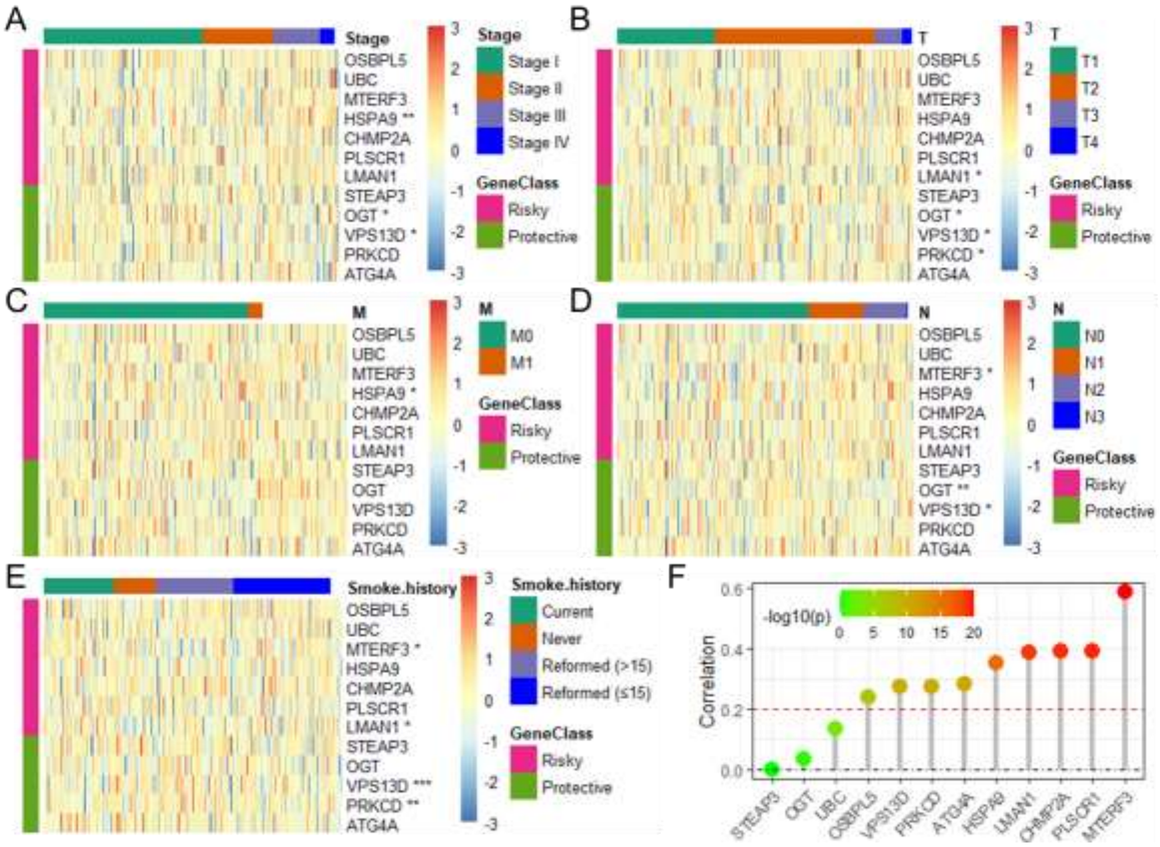


**Figure S4.** Expression of the 12 MRGs in TCGA-LUAD samples with different pathological features. Expression of the 12 MRGs in TCGA-LUAD samples with different stages (A), invasion depth (B), distant metastasis (C), lymph node metastasis (D), and smoking history. (F) correlation analysis between mitophagy risk score and the expression of 12 MRGs. TCGA: The Cancer Genome Atlas; LUAD: lung adenocarcinoma; MRGs: mitophagy related genes.

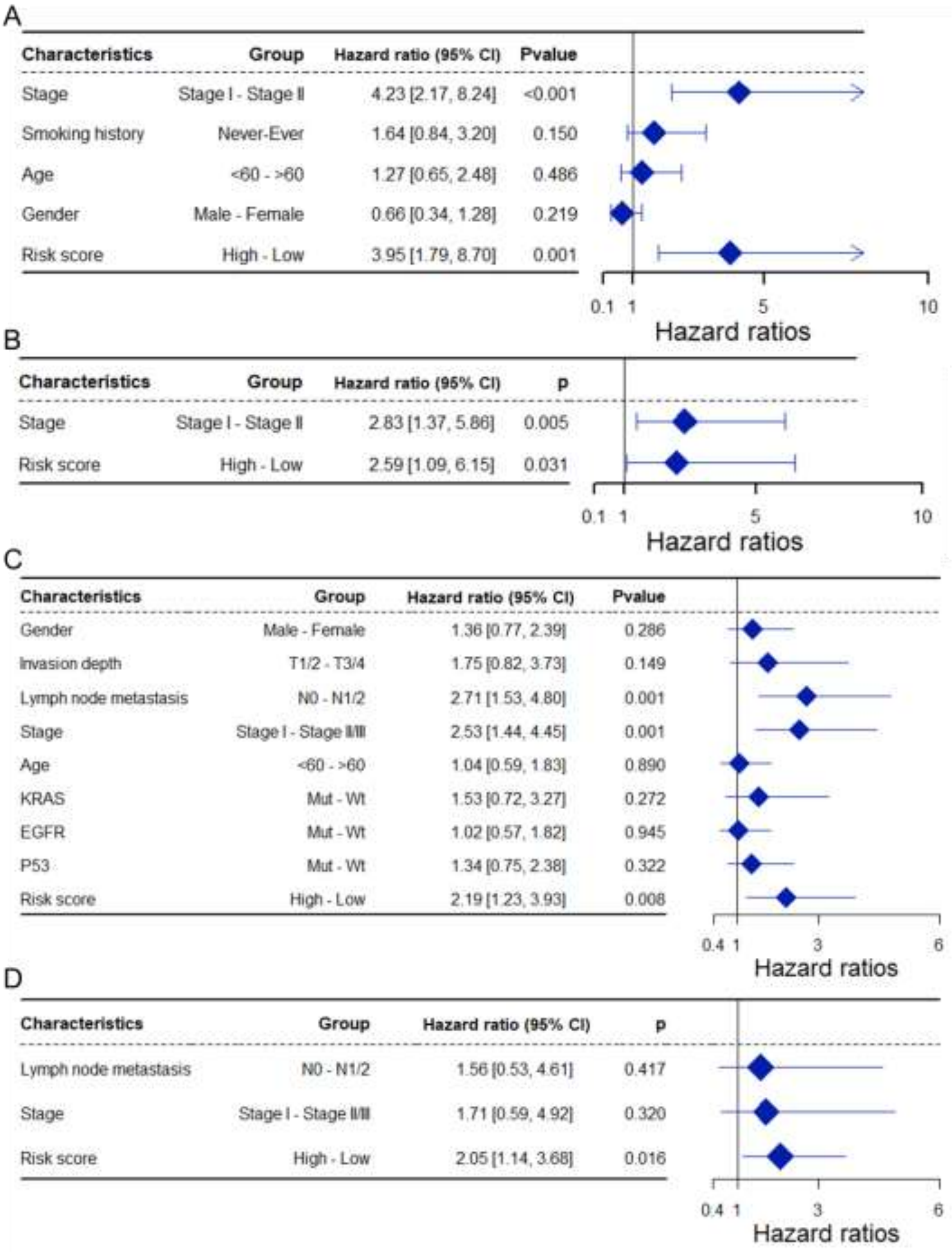


**Figure S5.** Mitophagy risk score is an independent prognostic factor for overall survival in validation datasets. Univariate (A) and multivariate (B) Cox regression analyses of risk score and clinic-pathological features for overall survival in GSE31210 dataset. Univariate (A) and multivariate (B) Cox regression analyses of risk score and clinic-pathological features for overall survival in GSE13213 dataset.

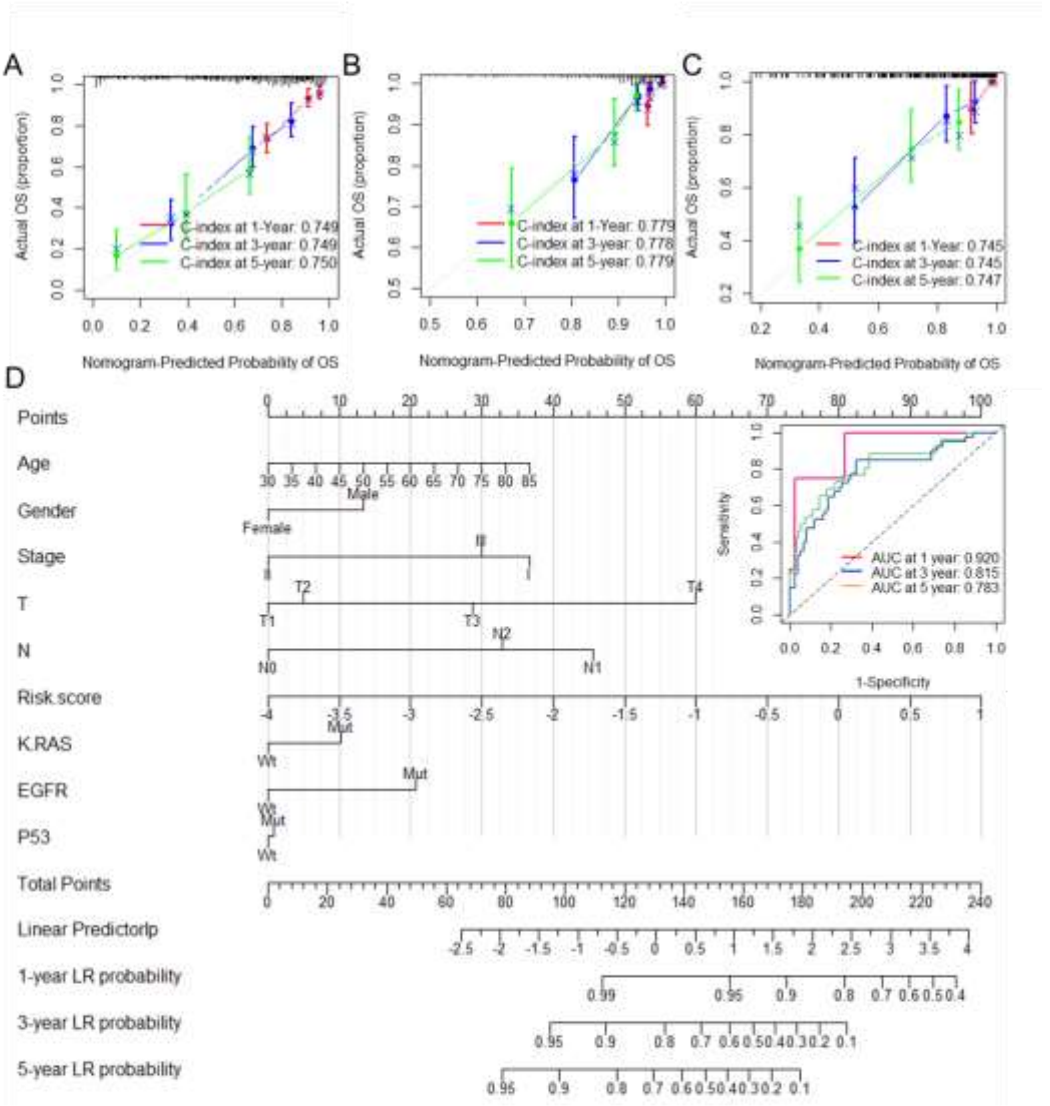


**Figure S6.** Nomogram predicting 1-year, 3-year and 5-year overall survival of lung adenocarcinoma patients. calibration plot for internal validation of the prognostic nomogram using TCGA-LUAD (A), GSE13213 (B) and GSE31210 (C) datasets. (D) The nomogram consists of the MRGs risk score and 8 clinical indicators based on the GSE31210 dataset. TCGA: The Cancer Genome Atlas; LUAD: lung adenocarcinoma; MRGs: mitophagy-related genes.

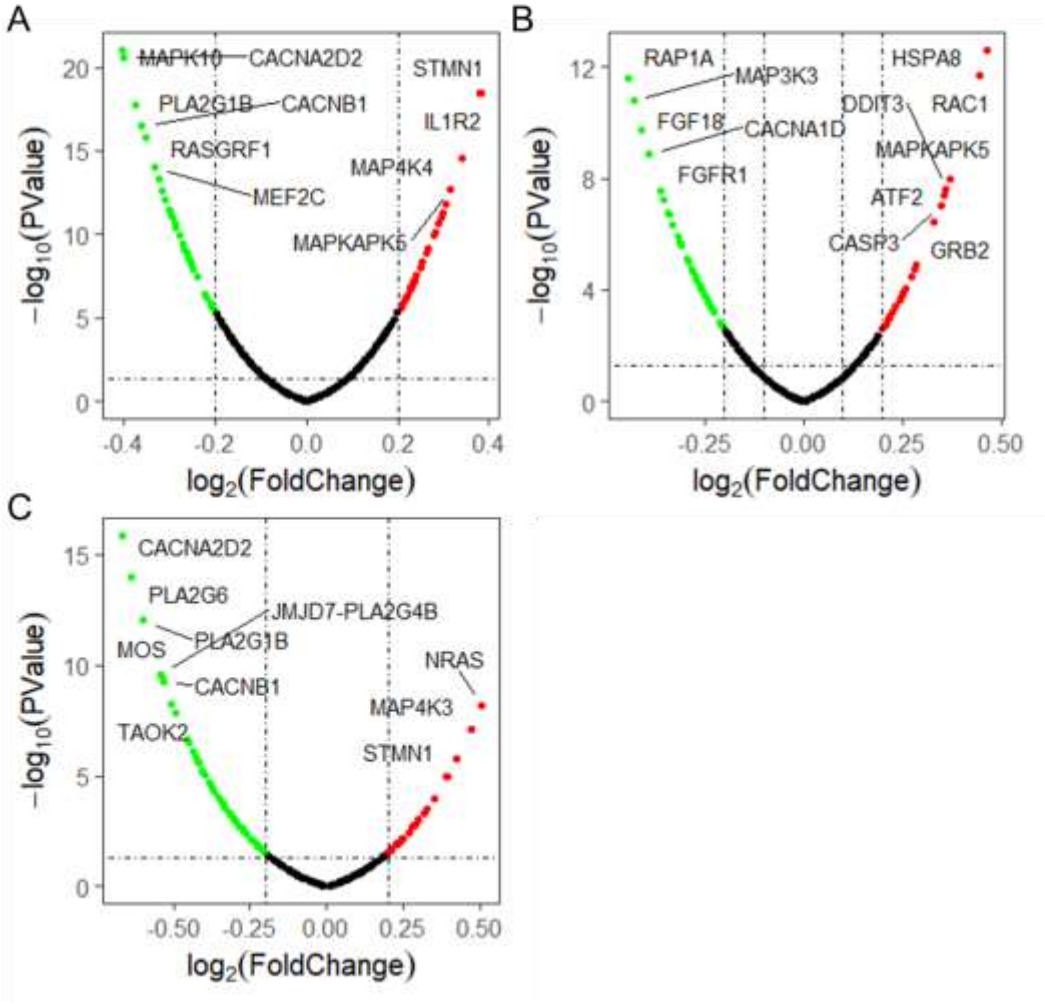


**Figure S7.** Volcano plots show the correlation between risk score and the expression of genes in the p38-MAPK pathway in TCGA-LUAD (A), GSE13213 (B) and GSE31210 (C) datasets. TCGA: The Cancer Genome Atlas; LUAD: lung adenocarcinoma.

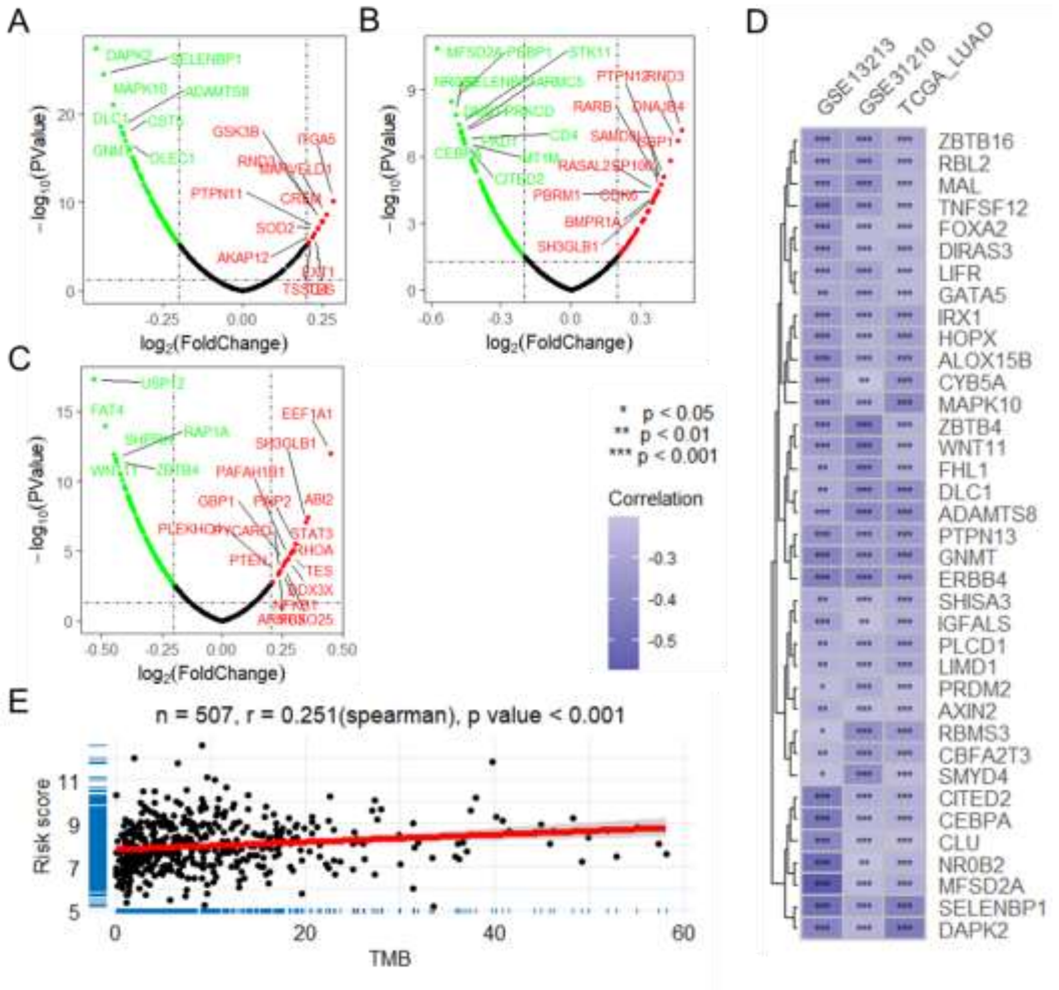


**Figure S8.** Mitophagy risk score is associated with tumor suppress genes expression and tumor mutation burden. Volcano plots show the correlation between risk score and the expression of tumor suppress genes in TCGA-LUAD (A), GSE13213 (B) and GSE31210 (C) datasets. (D) Heatmap shows the correlation between risk score and the expression of tumor suppress genes in 3 datasets. (E) scatter plot shows the correlation between risk score and tumor mutation burden. TCGA: The Cancer Genome Atlas; LUAD: lung adenocarcinoma.

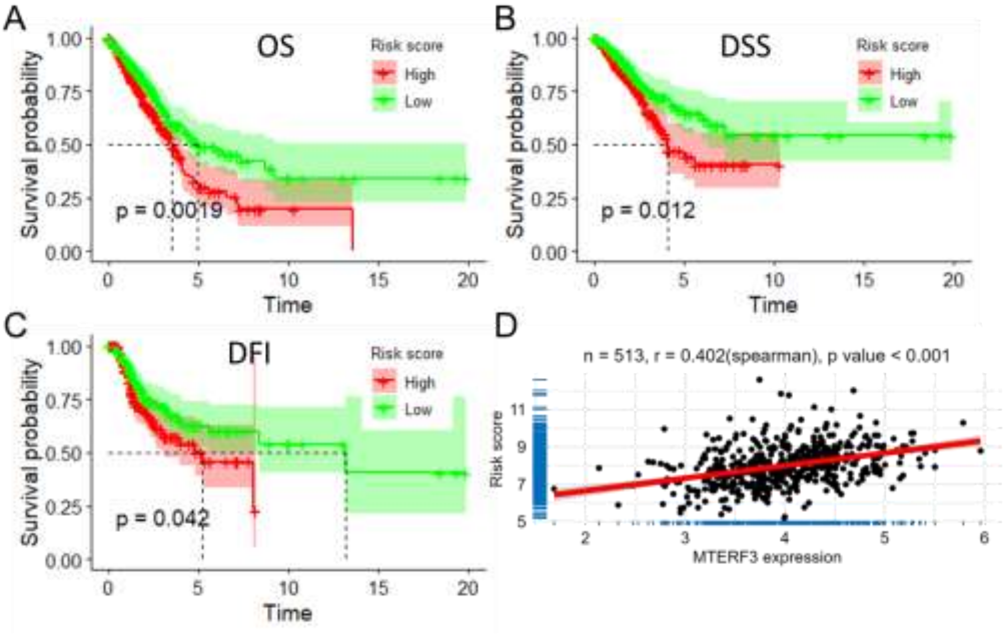


**Figure S9.** MTERF3 related to lung cancer prognosis. Kaplan–Meier curve of MTERF3 for OS (A), DSS (B), and DFI (C) in TCGA-LUAD dataset. (D) Scatter plot shows the correlation between risk score and MTERF3 expression. DSS, disease specific survival. DFI, disease free interval. OS, overall survival. LUAD, lung adenocarcinoma.

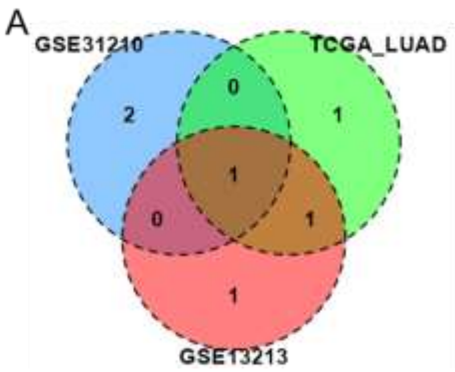


B

| GSE31210 | TCGA_LUAD | GSE13213 |
|---|---|---|
| UBC | MTERF3 | PLSCR1 |
| CHMP2A | HSPA9 | MTERF3 |
| **MTERF3** | LMAN1 | LMAN1 |

**Figure S10**. Identification of key MRG in the model. (A) The Venn diagram shows the intersection of the top three MRGs most correlated with risk scores in three datasets. (B) The table displays the top three MRGs most relevant to risk scores in each dataset. TCGA: The Cancer Genome Atlas; LUAD: lung adenocarcinoma; MRGs: mitophagy-related genes.

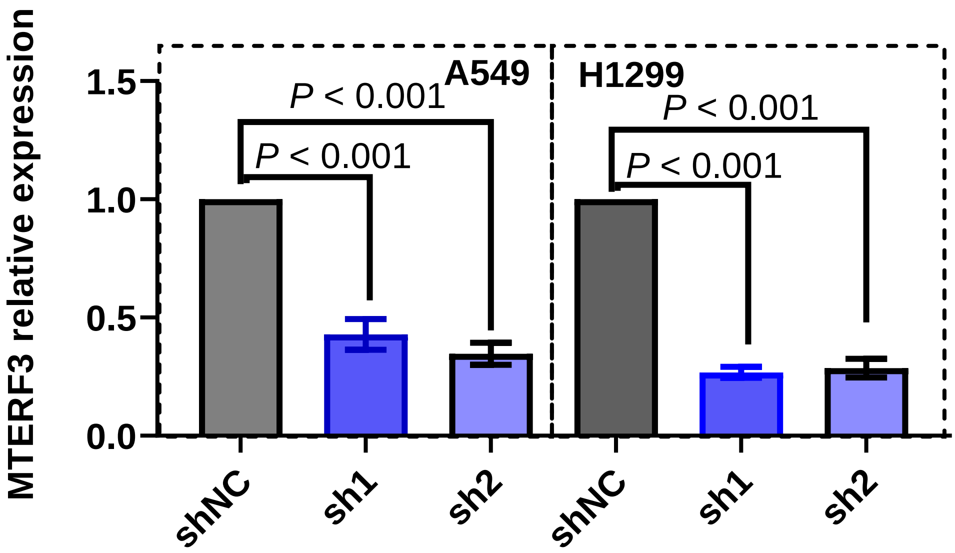


**Figure S11**. Validation of MTERF3 expression in LUAD cells transfected with MTERF3 knocked down plasmids.
